# Isolation and characterization of *Salmonella* phages for controlling bacterial infections

**DOI:** 10.3389/fvets.2025.1695255

**Published:** 2025-11-27

**Authors:** Seyyed Danial Mirmiran, Xinxin Li, Xiangmin Li, Ping Qian

**Affiliations:** 1 National Key Laboratory of Agricultural Microbiology, Hubei Hongshan Laboratory, Huazhong Agricultural University, Wuhan, Hubei, China; 2 College of Veterinary Medicine, Huazhong Agricultural University, Wuhan, Hubei, China; 3 Key Laboratory of Preventive Veterinary Medicine in Hubei Province, The Cooperative Innovation Center for Sustainable Pig Production, Wuhan, Hubei, China; 4 Hubei Jiangxia Laboratory, Wuhan, China

**Keywords:** *Salmonella enterica*, multidrug-resistant (MDR), phage, phage cocktails, phage therapy, biofilm

## Abstract

**Introduction:**

The rise of multidrug-resistant (MDR) *Salmonella enterica* poses a significant threat to public health, veterinary medicine, and food safety. Bacteriophages offer a promising alternative to antibiotics due to their host specificity and ability to lyse bacteria without disrupting commensal microbiota.

**Methods:**

In this study, twelve *Salmonella*-specific phages were isolated from diverse environmental sources across China. Specifically, DN01 originated from livestock manure in Shanxi Province (Northwest China), DN03 from poultry wastewater in Suizhou, Hubei Province (Central China), DN19 from slaughterhouse effluent in Guangdong Province (South China), and DN28 from hospital sewage in Zhejiang Province (Eastern China). This geographic and ecological diversity underscores the broad natural distribution of *Salmonella* phages, providing a representative foundation for subsequent morphological, genomic, and therapeutic analyses. The host bacterium *Salmonella Enteritidis* SE006, was used for phage propagation. Four lytic phages, which exhibited broad host ranges, were selected for in-depth characterization. Their biological properties, including optimal multiplicity of infection (MOI), latent period, burst size, pH, and thermal stability, and anti-biofilm activity, were systematically evaluated. Morphology was analyzed via transmission electron microscopy (TEM), and whole-genome sequencing, functional annotation, and phylogenetic analysis were conducted to assess genetic safety and taxonomic placement.

**Results:**

All phages exhibited potent lytic activity across multiple *Salmonella* serovars, including MDR strains, with MOIs as low as 0.00001 and short latent periods (10–20 min). They remained stable over a broad pH range (3–11) and exhibited thermal stability from 4 °C up to 50 °C, with partial loss of activity observed at 60 °C for some phages. Genomes ranged from 5,563 to 86,377 bp and lacked genes related to lysogeny, virulence, or antibiotic resistance. TEM and phylogenetic analyses classified the phages within distinct families of the Caudoviricetes class. In vitro assays demonstrated significant inhibition of bacterial growth and disruption of mature biofilms. Phage treatment significantly improved the survival of S. Enteritidis-infected mice over a 12-day period., survival rates were 0% (PBS), 25% (DN01), 33% (DN03), 75% (DN19), 85% (DN28), and 100% (cocktail).

**Conclusion:**

These results highlight the promise of DN01, DN03, DN19, and DN28, particularly in cocktail form, as safe, stable, and effective agents for phage-based control of multidrug-resistant *Salmonella enterica*.

## Introduction

*Salmonella enterica* is a major foodborne zoonotic pathogen, responsible for an estimated 153 million cases of gastroenteritis and over 57,000 deaths annually (WHO, 2022-DON369). It causes a wide spectrum of clinical outcomes in humans, ranging from self-limiting enteritis to severe systemic infections (1, 2). The primary transmission routes, contaminated food, water, and animal products, continue to pose significant challenges to food safety and public health systems worldwide (3–5). Recent findings have demonstrated the survivability of *Salmonella enterica* serovars under low-temperature food storage conditions, underscoring the importance of effective cold-chain management to prevent outbreaks (6).

A critical factor underlying *Salmonella*’s persistence is its ability to form biofilms, which enhance survival under hostile conditions and confer resistance to antibiotics and disinfectants (7–9). The escalating prevalence of multidrug-resistant (MDR) strains, particularly in Asia due to the misuse of antibiotics, underscores the urgent need for alternative antimicrobial strategies (10). Among these, phage-based approaches have emerged as a promising solution for biofilm control. Phages can specifically infect and lyse bacteria embedded in biofilms, degrade the extracellular polymeric matrix, and reduce bacterial load without affecting beneficial microbiota. Recent studies have demonstrated that both individual phages and phage cocktails are effective in preventing biofilm formation and eradicating established biofilms of *Salmonella* and other pathogens, highlighting their potential as innovative tools in combating biofilm-associated infections (11–13).

Lytic phages, in particular, have garnered renewed scientific interest as self-replicating, host-specific biocontrol agents with negligible impact on commensal microorganisms (14, 15). Recent studies have further emphasized their efficacy in disrupting biofilms formed by foodborne pathogens, highlighting their expanding role in food safety management (16). Their ability to combat multidrug-resistant (MDR) pathogens without contributing to antimicrobial resistance renders them highly suitable for food safety, veterinary, and clinical applications (17). Broad-host-range phages, in particular, hold considerable promise due to their capacity to target multiple *Salmonella* serovars.

Despite growing interest, many studies have limited their scope to host range or genomic features, often overlooking essential phenotypic and environmental analyses (18). Phages such as SHWT1 (19), STP4-a (20), and vB_SalP_LDW16 (21) have demonstrated considerable potential; however, more comprehensive evaluations are warranted, particularly in light of China’s diverse food ecosystems.

Temperate phages also play a key role in bacterial evolution by integrating as prophages and modulating virulence, antimicrobial resistance, and host fitness through horizontal gene transfer (22–25). Genomic surveys have identified hundreds of prophages across bacterial species (26).

In this study, four genetically safe, broad-host-range lytic phages against multidrug-resistant *Salmonella enterica* were isolated and characterized through *in vitro*, genomic, and *in vivo* murine assays. A phage cocktail showed synergistic antibacterial effects, highlighting their potential as effective agents for controlling MDR *Salmonella enterica* in veterinary and food safety applications. The use of a phage cocktail combines multiple lytic phages with complementary host ranges, enhancing therapeutic efficacy by targeting diverse *Salmonella* strains simultaneously. This strategy reduces the likelihood of resistant mutants emerging, ensures broader coverage, and provides more durable protection compared to individual phages. The superior survival and bacterial clearance observed in cocktail-treated mice in our study support the advantages of multi-phage formulations for controlling MDR *Salmonella* infections (15, 20). The experimental research results provide novel knowledge for bacterial evolution and raise public awareness for the prevention and control of *Salmonella*.

## Materials and methods

### Environmental sampling, isolation, and host range determination of *Salmonella* phages

Environmental samples, including sewage, poultry farm wastewater, slaughterhouse effluents from various Chinese provinces, and swine farm wastewater from Huizhou, were collected for the isolation of *Salmonella* phages. A total of 12 environmental samples were collected from diverse ecological niches across four provinces of China between January and June 2023, including livestock manure (Shanxi Province, Northwest China), poultry wastewater (Suizhou, Hubei Province, Central China), slaughterhouse effluent (Guangdong Province, South China), and hospital sewage (Zhejiang Province, East China). Using *Salmonella Enteritidis* SE006 obtained from the Key Laboratory of Huazhong Agricultural University as the host bacterium, four distinct lytic phages, DN01, DN03, DN19, and DN28, were successfully isolated. These isolates were selected for detailed characterization based on their broad host range, strong lytic activity, and high plaque clarity. Detailed information on all twelve initially isolated phages, including their isolation source, host range, plaque morphology, and selection criteria, is provided in (Supplementary Table S1).

The Samples were centrifuged (8,000–10,000 × *g*, 10 min) and filtered through a 0.22 μm membrane (Millipore) to remove debris and bacterial cells (27–29). Strain *S. enteritidis* SE006 was selected for *in vivo* studies due to its multidrug-resistant profile, clinical relevance, and well-characterized virulence in murine models. While the phages were tested against multiple *Salmonella* serotypes *in vitro*, SE006 served as a representative strain for *in vivo* evaluation, allowing assessment of therapeutic efficacy under controlled experimental conditions. This approach ensures that the results are both scientifically robust and translatable to controlling MDR *Salmonella* infections in real-world settings.

The filtrates were enriched with exponentially growing *Salmonella enterica*, primarily *S. Enteritidis* SE006 (GenBank: CP099973), in LB or TSB broth at 37 °C for 12–18 h with shaking. Bacterial cultures were grown in LB broth at 37 °C with orbital shaking at 180 rpm for 16 h. After centrifugation and filtration, lytic activity was evaluated using the double-layer agar method. Plaques were purified through three rounds of single-plaque isolation, and phages were subsequently amplified in SM buffer (Sodium-Magnesium buffer; 100 mM NaCl, 10 mM MgSO₄, 50 mM Tris–HCl, pH 7.5). High-titer lysates [>10^9^ plaque-forming units (PFU)/mL] were stored at 4 °C for short-term use or −80 °C in SM buffer supplemented with 20% glycerol (18).

Host range analysis was performed using *Salmonella* serovars *S. enteritidis, S. typhimurium, S. pullorum, S. Corvallis, S. Derby, S. Rissen, S. panama, S. Havana, S. Kentucky, S. Agona, S. India, S. typhi,* and *S. para-typhi.* Isolated from veterinary, clinical, and food sources. Strains were confirmed through biochemical testing and serotyping following national standards (10, 30).

### Host range analysis of *Salmonella* phages using spot assay and efficiency of plating (EOP)

The host range of *Salmonella*-specific phages was evaluated using spot assays and efficiency of plating (EOP) analysis. A diverse panel of *Salmonella enterica* strains, including clinically and epidemiologically relevant serovars such as *S. typhimurium, S. enteritidis,* and *S. Pullorum,* was tested. For the spot tests, high-titer phage lysates (~10^8^ PFU/mL) were applied to bacterial lawns prepared on tryptic soy agar (TSA) and incubated at 37 °C for 16–18 h. Clear zones were interpreted as strong lytic activity, while turbid zones indicated partial lysis (14, 19). Quantitative EOP assays were performed by spotting serially diluted phage suspensions onto the same strain panel. EOP was calculated as the ratio of plaque-forming units (PFU) on the test strain to those on the propagation host [The EOP = (PFUtest strain)/(PFUhost strainEC6) × 100%]. This combined approach provided a robust evaluation of each phage’s infectivity profile and lytic spectrum (21).

### Morphological analysis of *Salmonella* phages by transmission electron microscopy

The morphology of isolated *Salmonella* phages was examined via transmission electron microscopy (TEM) using negative staining. High-titer lysates (~10^9^–10^10^ PFU/mL) were purified by filtration and CsCl density gradient centrifugation. Purified phage suspensions were adsorbed onto carbon-coated copper grids, stained with 2% phosphotungstic acid, and visualized under a Hitachi H-7650 microscope at 80–120 kV. Adsorption to *S. enteritidis* was also assessed (14, 18).

### Determination of optimal multiplicity of infection (MOI) and one-step growth curve analysis of *Salmonella* phages

The optimal multiplicity of infection (MOI) for each *Salmonella*-specific phage was determined by infecting mid-log-phase *Salmonella enterica* cultures (OD₆₀₀ ≈ 0.4–0.6; ~10^7^ CFU/mL) with phage suspensions at MOIs ranging from 10^−7^ to 10. After 4–6 h of incubation at 37 °C with shaking, cultures were centrifuged, and supernatants were collected for plaque quantification using the double-layer agar method. The MOI that produced the highest phage yield was considered optimal and used in subsequent assays (18, 28, 31).

To evaluate phage replication dynamics, one-step growth curves were performed. Bacterial cultures were infected with phages at the optimal MOI and incubated for 5–10 min at 37 °C to allow initial phage–bacterium contact. Unbound phages were then removed by centrifugation and washing. The infected pellets were resuspended in fresh LB broth and incubated at 37 °C. Samples were collected at 10-min intervals up to 160 min, and phage titers were determined by the double-layer agar method. The latent period, rise phase, and burst size were calculated to characterize lytic kinetics and infection efficiency (20, 21, 32).

### Environmental stability assessment of *Salmonella* phages under varying pH and temperature conditions

The environmental stability of the four isolated *Salmonella* phages (DN01, DN03, DN19, and DN28) was assessed under a range of pH and temperature conditions, following procedures adapted from (33, 34).

For pH stability, phage lysates (10^8^–10^9^ PFU/mL) were incubated in SM buffer adjusted to pH 2.0–12.0 for 1 h at 37 °C. For thermal stability, phage suspensions were incubated at 4, 20, 37, 40, 45, 50, and 60 °C for 1 h. Following treatment, phage viability was determined using the double-layer agar plaque assay (35).

All experiments were performed in three independent biological replicates, each with three technical replicates. Data are reported as mean ± standard deviation (SD), and statistical analysis was conducted using GraphPad Prism 9.0 (GraphPad Software, San Diego, CA, USA). A *p*-value <0.05 was considered statistically significant.

### *In vitro* lytic activity of *Salmonella* phages assessed by optical density reduction

The lytic efficacy of isolated *Salmonella* phages was evaluated by monitoring bacterial growth inhibition at 600 nm (OD₆₀₀). Mid-log-phase *S. enterica* cultures (~10^7^ CFU/mL) were infected with phages at MOIs ranging from 0.0001 to 10 in 96-well microtiter plates and incubated at 37 °C for 12 h. OD₆₀₀ readings were recorded every 30–60 min using a microplate reader or Bioscreen C system. Non-infected bacteria and phage-only samples served as controls. A substantial decrease in OD₆₀₀ relative to the positive control indicated effective phage-induced lysis. This assay confirmed the strong antibacterial potential of the selected phages (19, 32).

### Anti-biofilm activity of *Salmonella* phages assessed by crystal violet staining and viable cell enumeration

The anti-biofilm properties of isolated *Salmonella* phages were evaluated by modified crystal violet (CV) staining. For inhibition assays, overnight *S. enterica* cultures were diluted 1:100 in half-strength LB and co-incubated with phages (10^7^ or 10^8^ PFU/mL) in 96-well plates at 30 °C or 37 °C for 24–48 h. To assess disruption, mature biofilms were pre-formed under similar conditions, rinsed with PBS, and treated with phages for 24 h. Residual biofilms were stained with 0.1–1% CV, solubilized with 95% ethanol, and quantified at 570 or 590 nm. Viable biofilm-associated bacteria were also enumerated by serial dilution and plating. All assays were conducted in triplicate, and results were statistically analyzed. Phages significantly inhibited biofilm formation and degraded established biofilms, supporting their potential for controlling *Salmonella* biofilms in clinical and veterinary settings (18, 21, 28).

### Genomic DNA extraction, whole-genome sequencing, annotation, and phylogenetic analysis of *Salmonella* phages

Genomic DNA of high-titer *Salmonella* phage lysates (10^9^–10^10^ PFU/mL) was extracted using commercial viral DNA extraction kits (Norgen Biotek, Canada; Omega Bio-Tek, USA). Lysates were pretreated with DNase I and RNase A to eliminate residual host nucleic acids. DNA quality and concentration were assessed using NanoDrop spectrophotometry (Thermo Fisher Scientific, USA) and 1% agarose gel electrophoresis.

Whole-genome sequencing was performed using the Illumina NovaSeq 6000 or MiSeq platforms (Novogene Bioinformatics Technology Co., Ltd., Beijing, China), generating 150 bp paired-end reads. Quality control of raw reads was assessed using FastQC v0.11.9 (Babraham Bioinformatics, Cambridge, UK), followed by adapter and low-quality base removal with Trimmomatic v0.39 (Bolger et al., 2014). *De novo* assembly was conducted using SPAdes v3.15.2 (36), and assembly quality was evaluated with QUAST v4.4 (37).

Genome annotation was performed using both the RAST server^1^ and Prokka v1.14.6 (Seemann, 2014), followed by manual curation with BLASTp against the NCBI non-redundant protein database.^2^ Transfer RNA (tRNA) genes were identified with tRNAscan-SE v2.0 (38).

To evaluate biosafety, phage genomes were screened for antibiotic resistance genes and virulence factors using the Comprehensive Antibiotic Resistance Database (CARD; https://card.mcmaster.ca/). The Virulence Factor Database (VFDB; http://www.mgc.ac.cn/VFs/). ResFinder^3^ and Virulence Finder.^4^ Analyses were conducted using default thresholds for all databases, with minimum identity and coverage values of 70%. Only hits meeting these criteria were retained. The database versions used were CARD (version 3.2.7) (39) and VFDB (updated 2024) (40). To avoid false positives, all identified ORFs were manually verified by BLASTp searches against the NCBI non-redundant protein database.

Genome termini and packaging strategies were inferred with PhageTerm v1.0.12 (Garneau et al., 2017). Comparative genomic analysis was conducted by aligning phage sequences with reference genomes using Easyfig v2.2.5 (41), and genome visualization was performed with CGView v2.0 (42). These analyses enabled the identification of conserved modules and unique genomic features among the isolates.

Phylogenetic relationships were determined based on the large terminase subunit gene or complete genome alignments. Reference genomes were retrieved following ICTV (International Committee on Taxonomy of Viruses) guidelines. Phylogenetic trees were constructed using MEGA v11.0 (43) with the neighbor-joining method and 1,000 bootstrap replicates to assess branch robustness.

### Evaluation of the therapeutic efficacy of *Salmonella* phages in a murine infection model animals and housing

Female BALB/c mice (6–8 weeks old, 20–25 g; *n* = 8 per group) were maintained under specific pathogen-free (SPF) conditions and acclimatized for 1 week prior to experimentation. All animal procedures were approved by the Scientific Ethics Committee of Huazhong Agricultural University (Approval ID: HZAUMO-2023-0007) and conducted in accordance with institutional ethical guidelines.

### Bacterial strain and infection

*Salmonella enterica* serovar Enteritidis SE006 was used for murine infection. Mice were intraperitoneally injected with 100 μL of bacterial suspension containing ~1 × 10^8^ CFU per mouse (1 × 10^9^ CFU/mL), a dose determined based on preliminary experiments and previously published murine models that reliably induce acute systemic infection without immediate lethality (19, 20).

### Phage preparation and treatment

Individual *Salmonella* phages (DN01, DN03, DN19, and DN28) were prepared at a titer of 1 × 10^10^ PFU/mL. For cocktail preparation, phages were mixed in equal titers to achieve equimolar contribution. Prior to *in vivo* administration, *in vitro* assays including pairwise and combined lytic activity tests were performed to exclude antagonistic interactions and confirm potential synergistic effects. The stability of the mixed cocktail was verified over 24 h at room temperature.

Mice received phage treatment 3–4 h post-infection to simulate early therapeutic intervention while allowing initial bacterial colonization. Control groups included PBS-treated mice, phage-only mice (no bacterial infection), and heat-inactivated phage or buffer-only controls for *in vitro* assays.

### Sample collection and bacterial phage quantification

Mice were monitored daily for 12 days for clinical signs, weight loss, and survival (35). At the end of the experiment or upon reaching humane endpoints, mice were euthanized via CO₂ inhalation. Organs including liver, spleen, cecum, and blood were aseptically harvested, homogenized, and analyzed for bacterial burden. Tissue samples were fixed in 10% neutral-buffered formalin, embedded in paraffin, sectioned, and stained with hematoxylin and eosin (H&E) for histopathological evaluation.

### Survival analysis

Survival outcomes were assessed using Kaplan–Meier analysis, and differences between groups were determined using the Log-rank (Mantel–Cox) test. A *p*-value <0.05 was considered statistically significant (GraphPad Prism version 9.0, GraphPad Software, San Diego, CA, USA). The 12-day observation period was chosen based on previous studies demonstrating sufficient duration to capture both acute and delayed mortality following phage therapy [(19–21); Li et al., 2021].

### Statistical analysis

All experiments, including *in vitro* host range determination, lytic activity assays, one-step growth curves, biofilm inhibition/disruption assays, and thermal and pH stability tests, were performed with at least three independent biological replicates, each consisting of three technical replicates. Data are presented as mean ± standard deviation (SD). Graphical representations include error bars indicating SD, and the number of replicates is specified in figure legends.

## Results

### Host range and lytic spectrum of *Salmonella* phages against diverse serotypes

**Figure 1:**
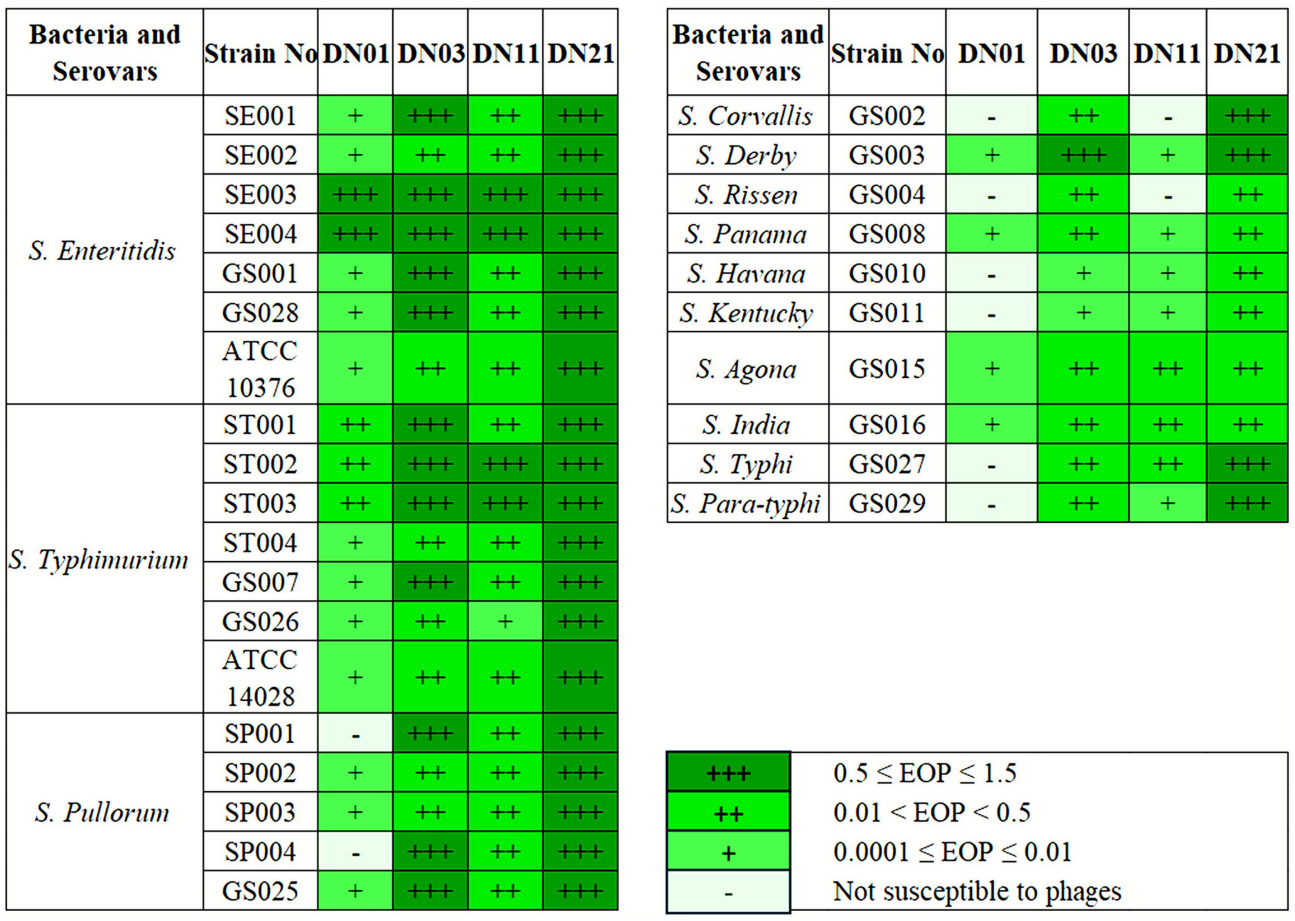
Host range and lytic spectrum of the selected *Salmonella* phages DN01, DN03, DN19, and DN28. The four phages were selected from twelve initially isolated bacteriophages based on their broad host range and high plaque clarity observed during preliminary screening (see Supplementary Table S1). Their lytic activity was evaluated against 29 *Salmonella enterica* strains representing diverse serovars and geographic origins. DN01 lysed 21 strains (72.4%), DN19 lysed 27 strains (93.1%), whereas DN03 and DN28 lysed all tested strains (100%), demonstrating broad host adaptability and strong lytic potential. Comparative analysis showed significantly higher lytic efficiency for DN03 and DN28 (*p* < 0.01). Data represent the mean ± standard deviation from three independent experiments.

The host range of the four *Salmonella* phages (DN01, DN03, DN19, and DN28) was evaluated against 29 strains representing diverse serovars and geographic origins. DN01 lysed 21 strains (72.4%), DN19 lysed 27 strains (93.1%), while DN03 and DN28 lysed all 29 strains (100.0%), indicating broad host adaptability (Figure 1). Comparative analysis showed higher lytic efficiency for DN03 and DN28 compared to DN01 (*p* < 0.01), whereas DN19 exhibited slightly delayed plaque formation. The lytic activity was determined based on plaque clarity and diameter using a spot assay, as detailed in the Materials and Methods section. Detailed host range profiles of all 12 initially isolated phages are provided in (Supplementary Table S1).

### Morphological characterization of *Salmonella* phages by plaque assay and transmission electron microscopy

**Figure 2:**
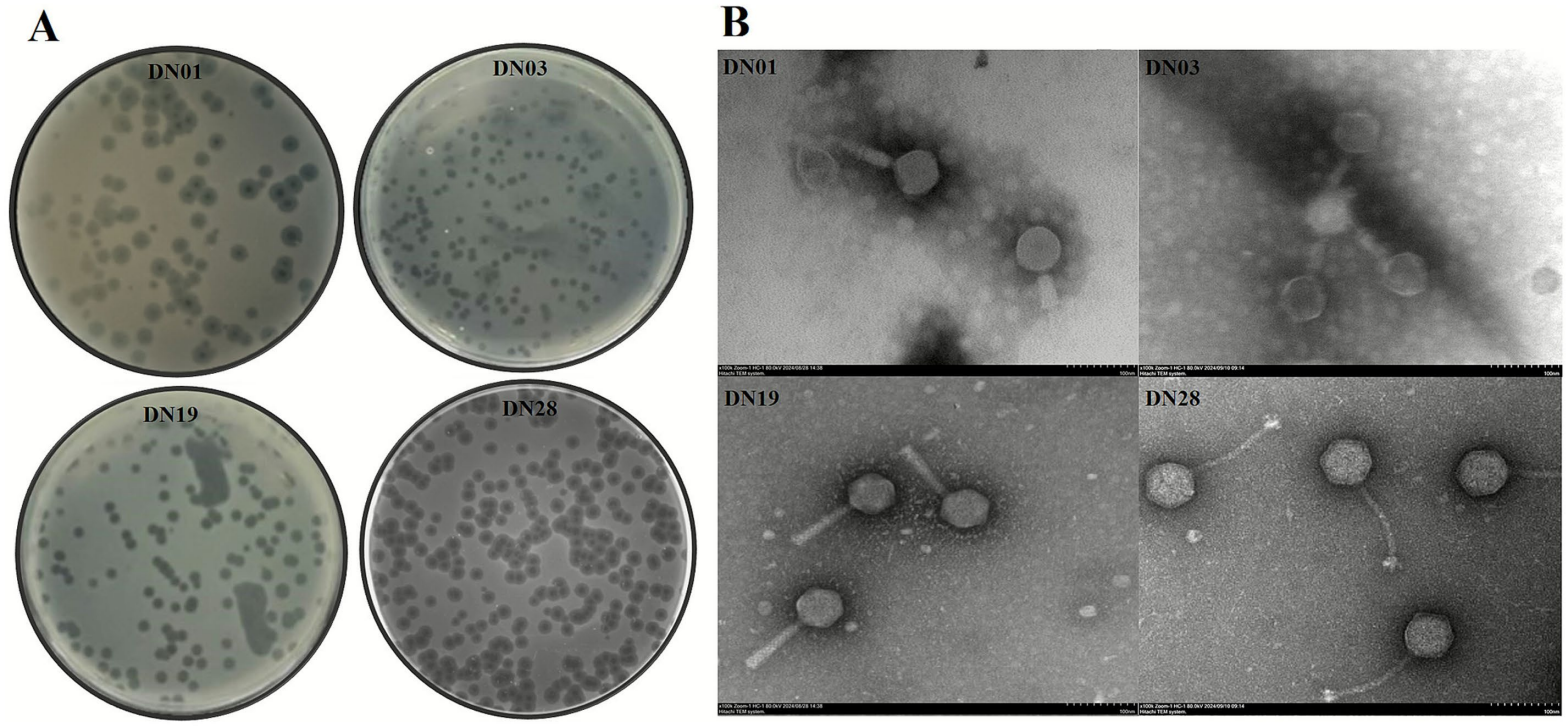
Plaque Morphology and TEM of *Salmonella* Phages. **(A)** Plaque morphology of *Salmonella* phages DN01, DN03, DN19, and DN28 on *Salmonella enterica* lawns. **(B)** Transmission electron micrographs of the four phages after negative staining with phosphotungstic acid (PTA). The measured head diameters and tail lengths are indicated in the Results section.

Plaque morphology analysis (Figure 2A) revealed that all four *Salmonella* phages produced clear, round plaques with diameters ranging from 1.5 to 3 mm, indicative of strong lytic activity. Transmission electron microscopy (TEM) with negative staining using 2% phosphotungstic acid (PTA) demonstrated that the four phages exhibited distinct morphological types corresponding to their genomic classification (Figure 2B). Phage DN01 (Demerecviridae) displayed an icosahedral head (~75 ± 5 nm in diameter) with a short, noncontractile tail (~20 ± 3 nm), consistent with T5-like morphology. DN03 (Drexlerviridae) possessed a long, flexible, noncontractile tail (~180 ± 10 nm) attached to an isometric head (~60 ± 4 nm), characteristic of T1/T5-like siphoviruses. DN19 (Autographiviridae) had a small icosahedral head (~55 ± 3 nm) and a short tail (~15 ± 2 nm), resembling T7-like podoviruses. DN28 (Straboviridae) exhibited a large icosahedral capsid (~90 ± 6 nm) and a long, contractile tail (~110 ± 8 nm), typical of T4-like myoviruses. These morphological distinctions are consistent with the family-level classification determined by genomic and phylogenetic analyses, supporting their taxonomic placement within Caudoviricetes.

### Determination of optimal multiplicity of infection (MOI) for efficient replication of *Salmonella* phages

**Figure 3:**
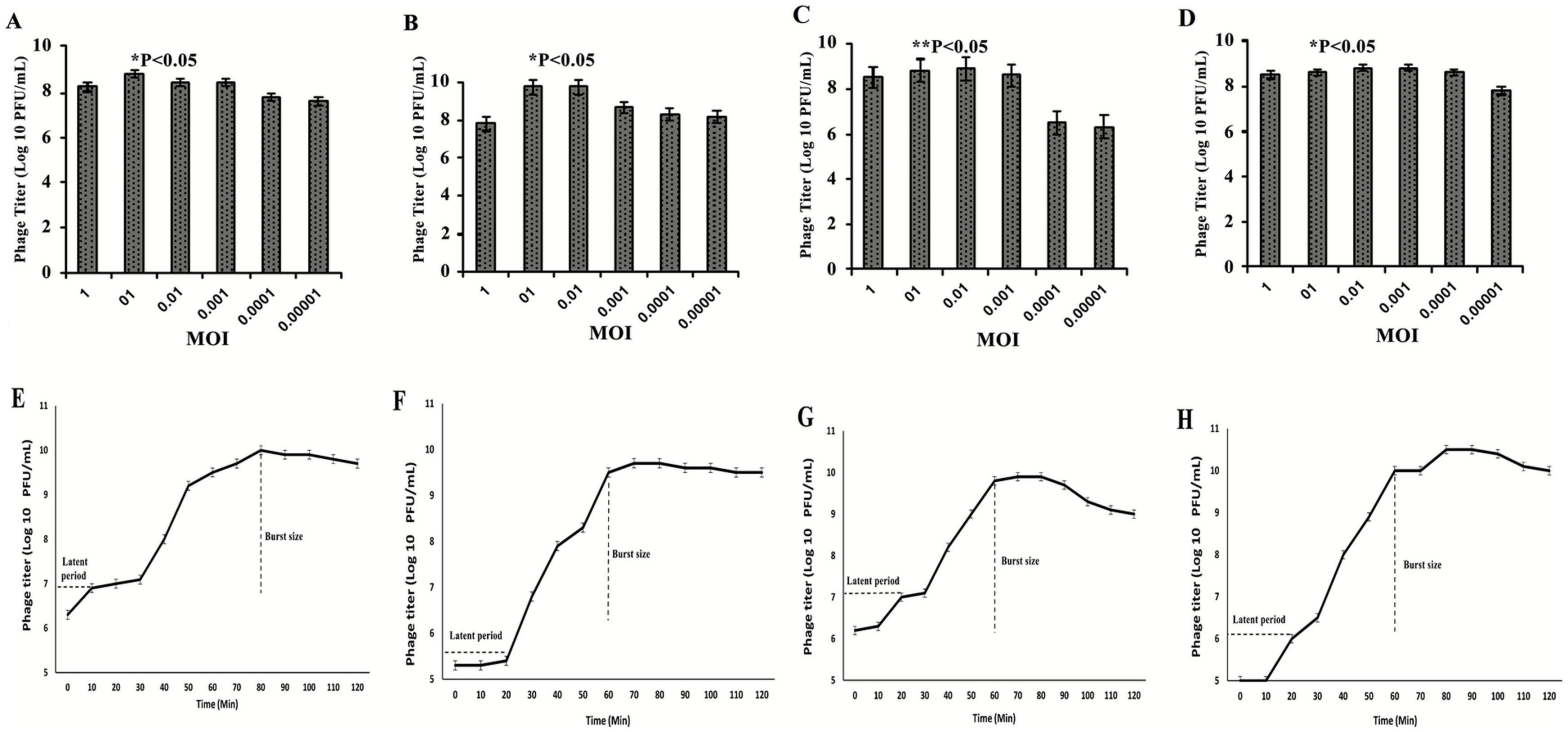
Optimal multiplicity of infection (MOI) and one-step growth kinetics of four lytic *Salmonella* phages. **(A–D)** Optimal MOI determination based on the highest phage yield after 4 h incubation with *S. enteritidis* SE006. **(E–H)** One-step growth curves showing latent periods, rise phases, and burst sizes of the phages over 160 min. Data represent mean ± SD from triplicate experiments. Statistical analysis was performed using one-way ANOVA with Tukey’s *post hoc* test; *p* < 0.05, *p* < 0.01 vs. MOI = 1.

The optimal multiplicity of infection (MOI) for each phage was determined by infecting host bacteria with serially diluted phage suspensions (MOIs 1–0.00001) and measuring titers after incubation (Figures 3A–D). DN01 and DN03 reached maximum yields at an MOI of 0.1, while DN19 and DN28 peaked at 0.01. Notably, DN19 and DN28 maintained high replication even at very low MOIs (≤0.0001), indicating robust propagation under limited host availability. These values represent optimal MOIs for phage amplification *in vitro* and should not be taken as optimal ratios for bacterial inhibition, which depend on factors such as adsorption rate, lysis time, and host physiological state.

### One-step growth curve analysis and replication kinetics of *Salmonella* phages

The replication kinetics of the four *Salmonella* phages are illustrated in (Figures 3E–H). DN01 exhibited a latent period of approximately 10 min, followed by a rise phase of about 80 min before reaching the plateau stage. In contrast, DN03, DN19, and DN28 showed latent periods of around 20 min, with rise phases lasting approximately 60 min. The observed infection dynamics demonstrate consistent and reproducible replication profiles among the tested lytic phages.

Based on the one-step growth curve data, the calculated burst size of DN01 was approximately 80 PFU per infected cell, whereas DN03, DN19, and DN28 each exhibited burst sizes of about 60 PFU per infected cell. These values fall within the expected range reported for *Salmonella*-infecting lytic phages, confirming their efficient replication cycles and robust lytic potential. The relatively shorter latent period and higher burst size observed for DN01 suggest a faster replication rate and potentially stronger lytic activity compared to the other three phages.

Collectively, these findings indicate that all four phages exhibited efficient propagation characteristics, with DN01 showing slightly superior replication kinetics under the tested conditions.

### Stability of *Salmonella* phages under varying pH and temperature conditions

**Figure 4:**
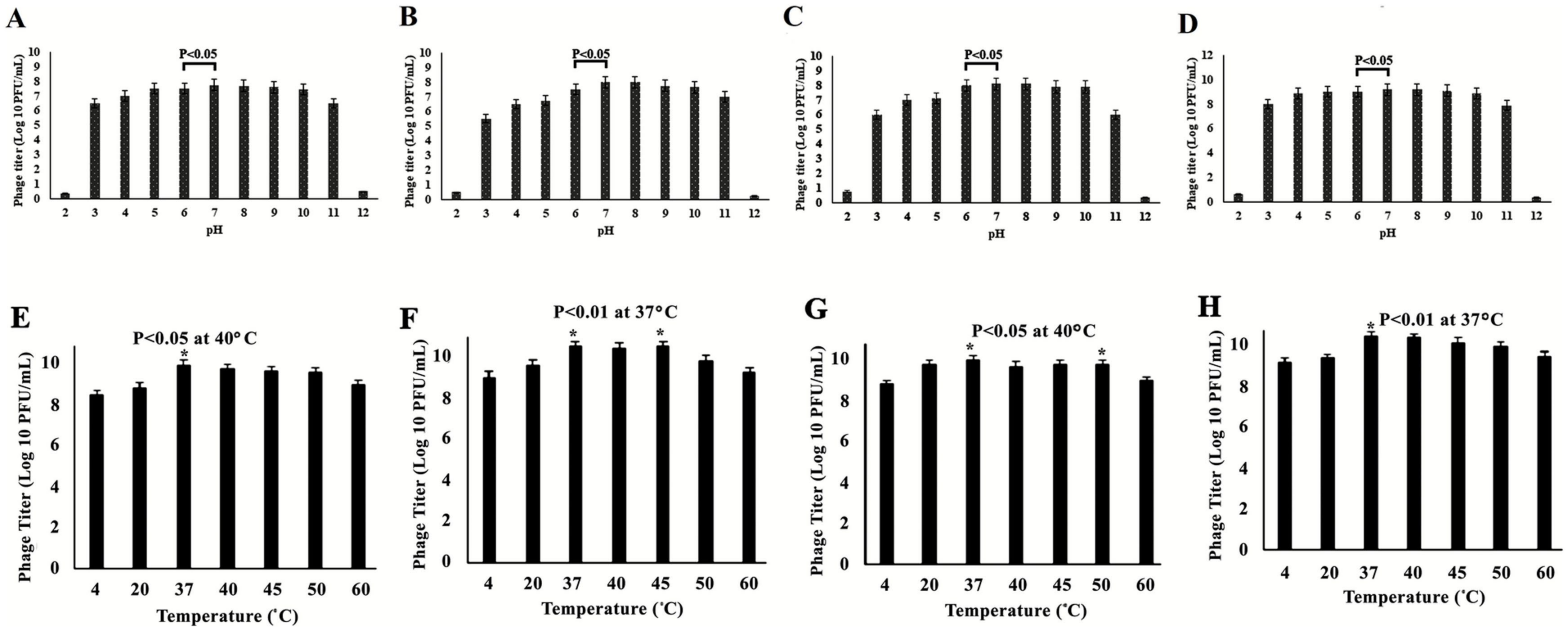
Environmental stability of selected *Salmonella* phages under varying pH and temperature conditions. **(A–D)** Phage stability following 1 h incubation across a pH range of 2–12, measured by log₁₀ PFU/mL titers. All four phages retained high stability at neutral to mildly alkaline pH (7–9), with significant reductions in activity observed under strongly acidic (pH 2–3) and basic (pH 11–12) conditions (*p* < 0.05). **(E–H)** Thermal stability of phages incubated at 4 °C to 60 °C. Peak titers were observed between 37 °C and 45 °C, indicating optimal stability within physiological temperatures, whereas reduced viability occurred at lower (4 °C) and higher (60 °C) extremes (*p* < 0.05; *p* < 0.01). Data represent mean ± SD (*n* = 3). These results support the potential of the phages for gastrointestinal or clinical applications requiring environmental resilience.

The environmental stability of four *Salmonella* phages (DN01, DN03, DN19, DN28) was evaluated across pH 2–12 to assess their practical and therapeutic potential (Figures 4A–D). Phage titers (log₁₀ PFU/mL) were measured after 1 h at each pH, with results as mean ± SD (0.3) of three experiments. All phages remained stable at neutral to mildly alkaline pH (7–9) and retained infectivity from pH 3–11. Significant titer reductions occurred at pH < 3 or >11 (*p* < 0.05), emphasizing the influence of environmental pH on phage stability.

Thermal stability assays (Figures 4E–H) revealed that phage DN01 maintained high infectivity up to 50 °C, with minor reduction thereafter. DN19 exhibited similar tolerance but declined sharply at 60 °C. DN03 and DN28 were more heat-sensitive; DN03 lost most activity ≥60 °C, while DN28 remained stable at 50 °C with moderate reduction at 60 °C. Overall, all phages remained stable up to ~50 °C, supporting their potential for practical applications.

Collectively, these findings demonstrate the robust physicochemical stability of the four *Salmonella* phages under neutral to moderately stressful conditions, supporting their potential application in oral delivery systems, veterinary formulations, and food-safety interventions where pH and temperature fluctuations are common.

### *In vitro* lytic activity of *Salmonella* phages against *Salmonella enterica* SE006 at varying MOIs

All four phages exhibited strong lytic activity against *Salmonella enterica* SE006 across a broad range of multiplicities of infection (MOIs; 0.0001–10), as indicated by an initial decrease in OD₆₀₀ followed by partial bacterial regrowth. It is important to clarify that the term “resistant mutants” in this context refers exclusively to bacterial subpopulations that developed resistance to phage infection, rather than to antibiotic resistance. No antibiotic susceptibility testing of SE006 was conducted in this study.

**Figure 5:**
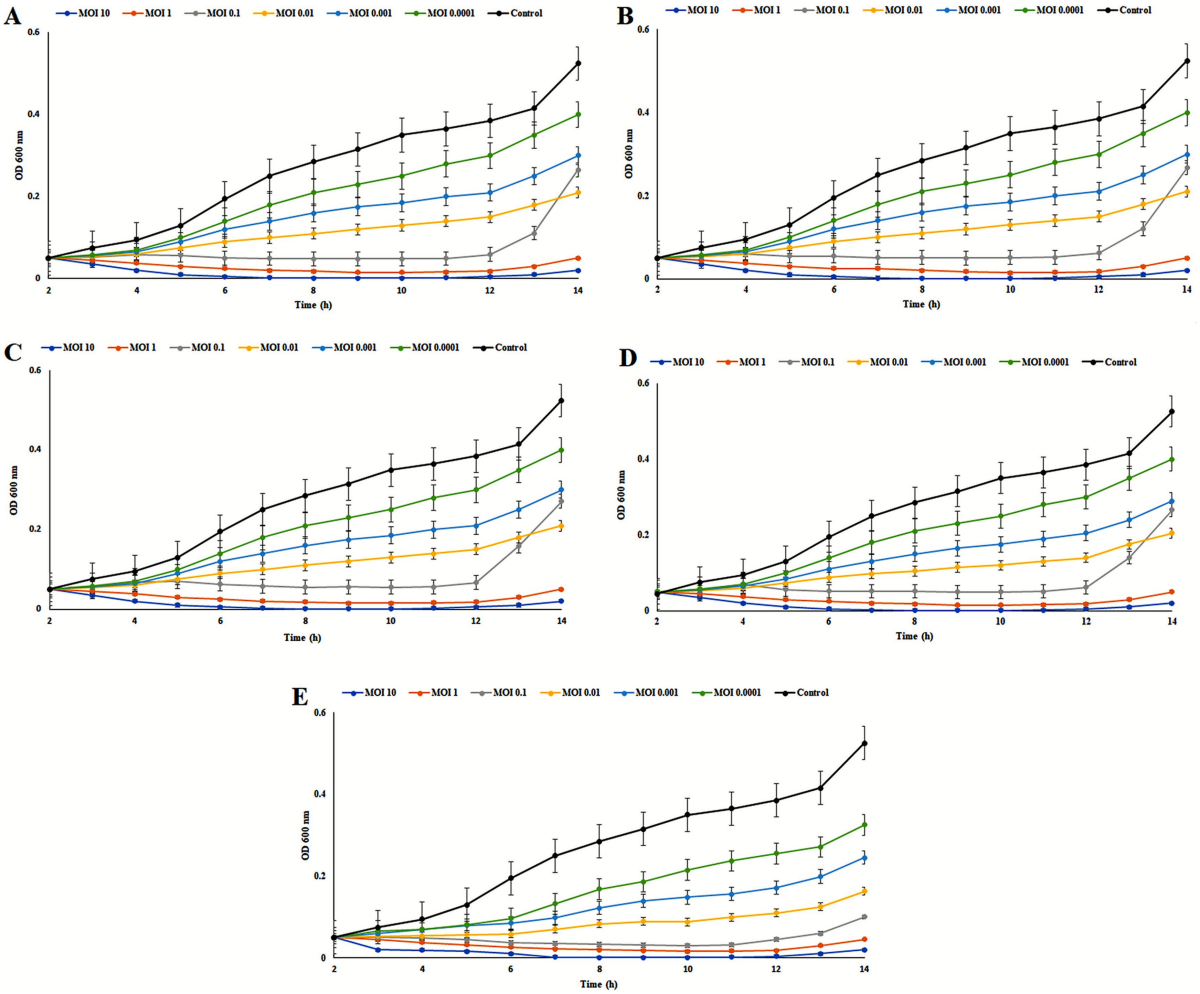
Lytic activity of four isolated *Salmonella* phages **(A)** DN01, **(B)** DN03, **(C)** DN19, **(D)** DN28 and their receptor-guided (E) cocktail against *S. enterica* SE006 at different multiplicities of infection (MOIs). Bacterial cultures at mid-log phase (OD₆₀₀ ≈ 0.5) were infected with phage suspensions at MOIs ranging from 0.0001 to 10, and bacterial growth was monitored by measuring optical density at 600 nm (OD₆₀₀) at regular intervals over time (h). Each curve represents the mean ± standard deviation (SD) of three independent biological replicates. All phages exhibited rapid bacterial lysis during the early infection phase, followed by partial regrowth attributed to the emergence of phage-resistant subpopulations. The receptor-guided phage cocktail showed superior lytic efficacy and delayed bacterial regrowth compared with single-phage treatments.

Higher MOIs resulted in more rapid and extensive bacterial lysis, while the receptor-guided phage cocktail exhibited superior efficacy in suppressing bacterial regrowth compared with individual phage treatments (Figure 5). This enhanced performance reflects the broader host range and complementary receptor usage of the combined phages.

Collectively, these findings demonstrate the potent bacteriolytic activity of the isolated phages and the improved effectiveness of the multi-phage cocktail. The results also emphasize the importance of distinguishing phage-resistance phenomena from antibiotic resistance when interpreting bacterial regrowth dynamics in lysis assays.

### Phage-mediated inhibition and disruption of *Salmonella enterica* SE006 biofilms

**Figure 6:**
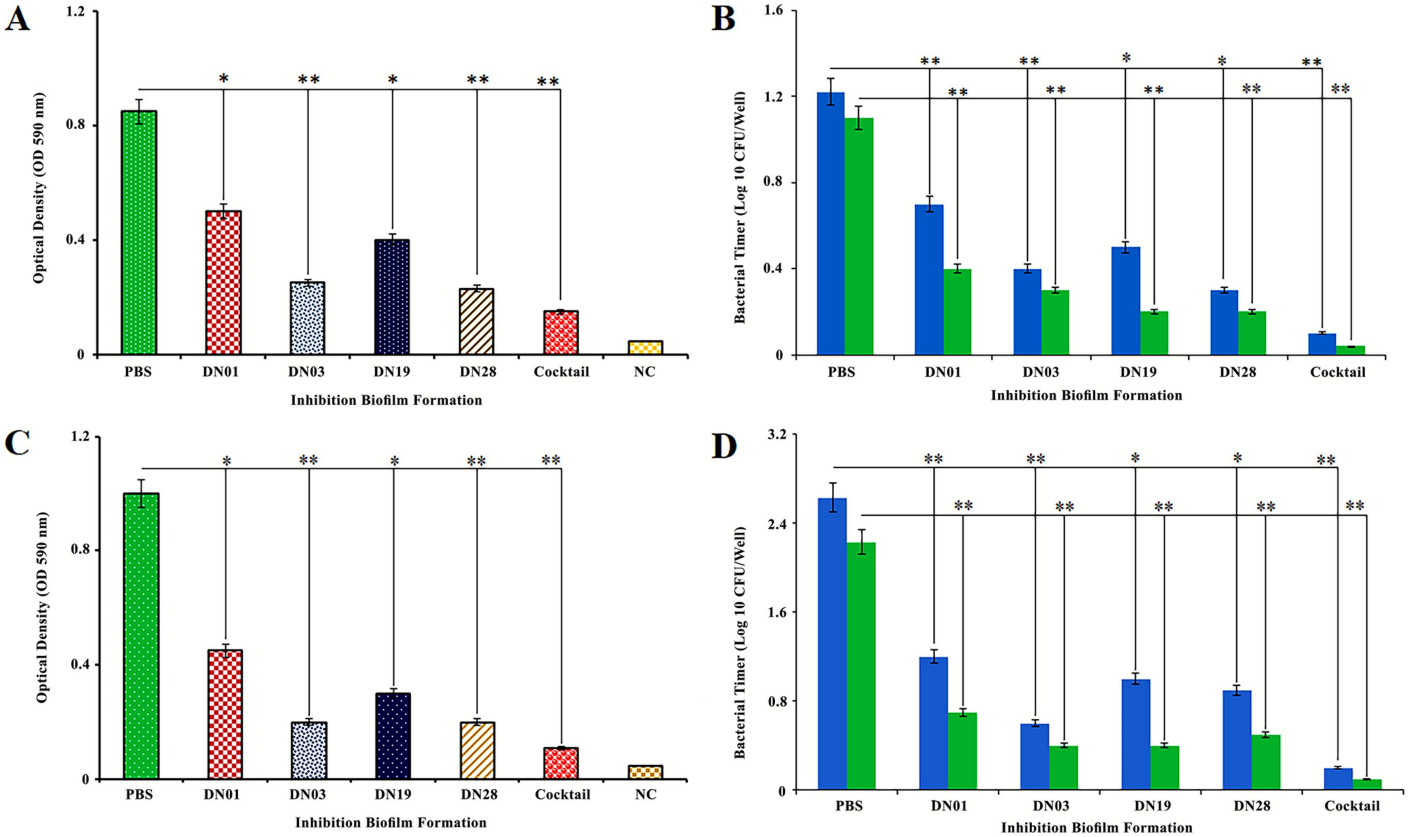
Inhibition and disruption of *Salmonella Enteritidis* SE006 biofilms by four lytic phages. **(A, B)** Biofilm formation inhibition after 24 h and 48 h co-incubation of SE006 with phages DN01, DN03, DN19, and DN28 (10^8^ PFU/mL). **(C, D)** Disruption of pre-formed biofilms following 24 h phage treatment at 37 °C. Biofilm biomass was quantified by crystal violet staining **(A, C)**, and viable cell counts were determined by plating assays **(B, D)**. Data are presented as mean ± SD (*n* = 3). Statistical significance: *p* < 0.05, *p* < 0.01 (one-way ANOVA with post hoc analysis).

The anti-biofilm efficacy of four lytic *Salmonella* phages (DN01, DN03, DN19, and DN28) was evaluated against *Salmonella enterica* SE006 using a 96-well microtiter plate assay. Treatment with 10^8^ PFU/mL significantly inhibited biofilm formation during a 48-h incubation, as determined by optical density (OD₅₉₀) and viable-cell enumeration (Figure 6). The inhibition was dose-dependent, with higher phage titers producing greater reductions in biofilm biomass and cell viability.

To assess eradication capacity, mature biofilms established over 48 h were exposed to the same phage concentration for 24 h. Both OD₅₉₀ and viable-cell counts indicated marked biofilm degradation, demonstrating that these phages effectively prevent and disrupt biofilms.

Among individual phages, DN19 and DN28 showed the strongest effects, reducing biofilm biomass by approximately 68 and 72%, respectively, whereas DN01 and DN03 achieved 55 and 63% reductions. A receptor-guided cocktail containing all four phages achieved nearly 89% inhibition of biofilm formation and 90% disruption of mature biofilms, indicating strong synergistic activity.

These findings highlight the robust anti-biofilm potential of the four *Salmonella* phages and support their use as promising biocontrol agents for managing biofilm-associated infections in veterinary and food safety contexts.

### Genomic features and phylogenetic classification of *Salmonella* phages

**Figure 7:**
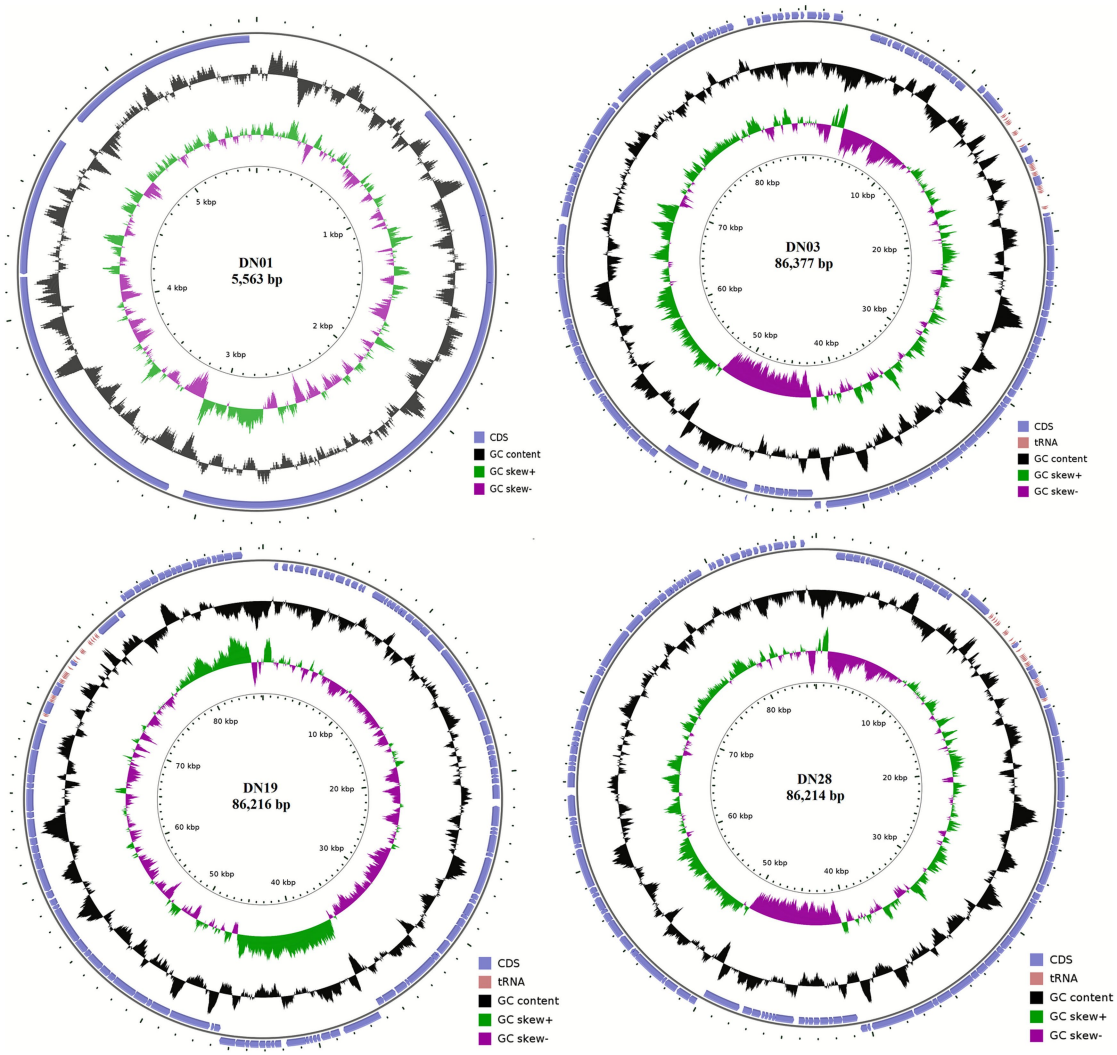
The genomic architectures of the four phages are illustrated, showing annotated functional modules including replication, structural, and lysis genes. DN01 harbors a compact 5,563 bp dsDNA genome (GC content 46.8%), whereas DN03, DN19, and DN28 possess genomes of approximately 86 kb. Despite its unusually small genome, DN01 exhibits functional lysis genes and clear plaque morphology, confirming its biological validity as a *Salmonella*-associated phage. Phylogenetic analysis classified the phages into families Demerecviridae (DN01, T5-like), Drexlerviridae (DN03, T1/T5-like), Autographiviridae (DN19, T7-like), and Straboviridae (DN28, T4-like) following (46) guidelines. Bootstrap values ≥86% indicate robust family-level assignments. DN01 occupies an intermediate position relative to other Salmonella and *E. coli* phages, suggesting potential cross-genus host-range overlap. The phylogenetic topology is consistent with whole-genome BLAST comparisons and functional gene content, confirming distinct evolutionary lineages among the four phages (47, 53).

Genomic characterization provides essential insights into the safety, replication dynamics, and host specificity of phages intended for therapeutic applications. Whole-genome sequencing revealed that the four *Salmonella*-specific phages possessed linear double-stranded DNA (dsDNA) genomes of distinct sizes: DN01 (5,563 bp; GC content 46.8%), DN03 (86,377 bp), DN19 (86,216 bp), and DN28 (86,214 bp) (Figure 7). No lysogeny-associated, virulence-related, or antibiotic resistance genes were detected, confirming their genomic safety and suitability for therapeutic use (44, 45). Multiple lysis-related genes were identified, including lysozyme in DN01, *holin* in DN03, and cell wall hydrolases in DN19 and DN28, which correlate with the strong bacteriolytic activity observed *in vitro*.

**Figure 8:**
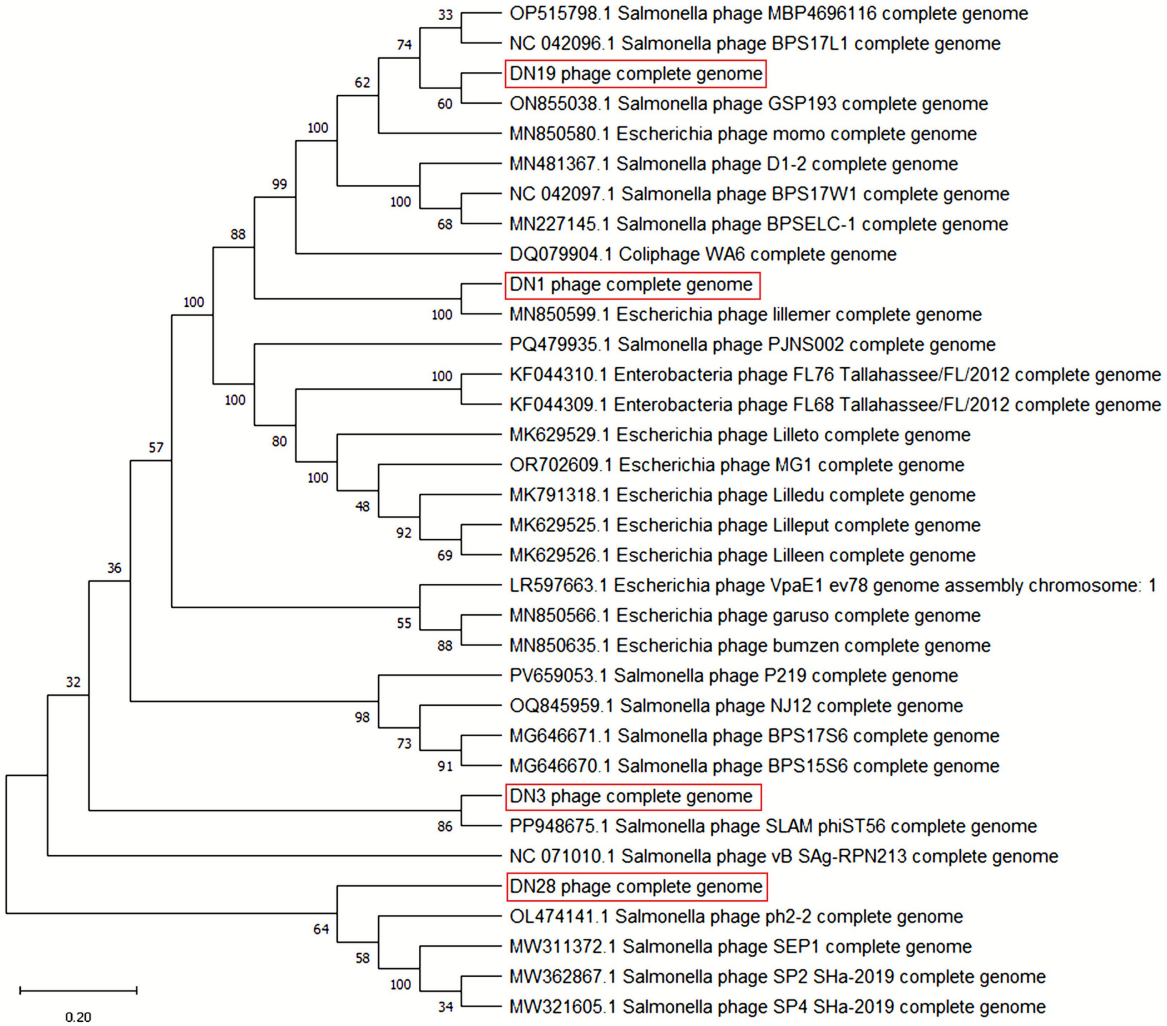
Phylogenetic analysis of *Salmonella* phages. Whole-genome-based maximum-likelihood phylogenetic tree depicting the relationships of *Salmonella* phages DN01, DN03, DN19, and DN28 with representative members of the class Caudoviricetes. Bootstrap values ≥30% are shown at branch nodes. Viral families are designated according to the latest ICTV taxonomy: Autographiviridae (T7-like), Demerecviridae (T5-like), Drexlerviridae (T1/T5-like siphoviruses), and Straboviridae (T4-like). DN phages are highlighted in bold. The scale bar indicates substitutions per site.

DN01, DN03, DN19, and DN28 were taxonomically assigned to the families Demerecviridae, Drexlerviridae, Autographiviridae, and Straboviridae, respectively, following ICTV (46) criteria (Figure 8). Bootstrap values ≥86% supported these family-level assignments, which were also consistent with the phages’ morphological characteristics. Methodological details of the phylogenetic and genomic analyses are provided in the Materials and Methods section.

Notably, DN01 exhibited a remarkably smaller genome compared to typical Caudoviricetes members. Its compact genomic architecture, characterized by overlapping and densely arranged open reading frames (ORFs), resembles single-stranded DNA phages such as Microviridae and Inoviridae, which are known for rapid infection cycles in enteric hosts (47, 48). This compact structure may contribute to the rapid replication cycle and enhanced lytic efficiency of DN01, suggesting potential host-range overlap between *Salmonella* and *E. coli*, although its classification remains as a *Salmonella* phage. While technical artifacts (e.g., sequencing errors) cannot be completely ruled out, multiple lines of evidence, including ORF organization and functional modules, support the authenticity of DN01.

### Therapeutic efficacy of *Salmonella* phages and phage cocktail in a murine infection model

**Figure 9:**
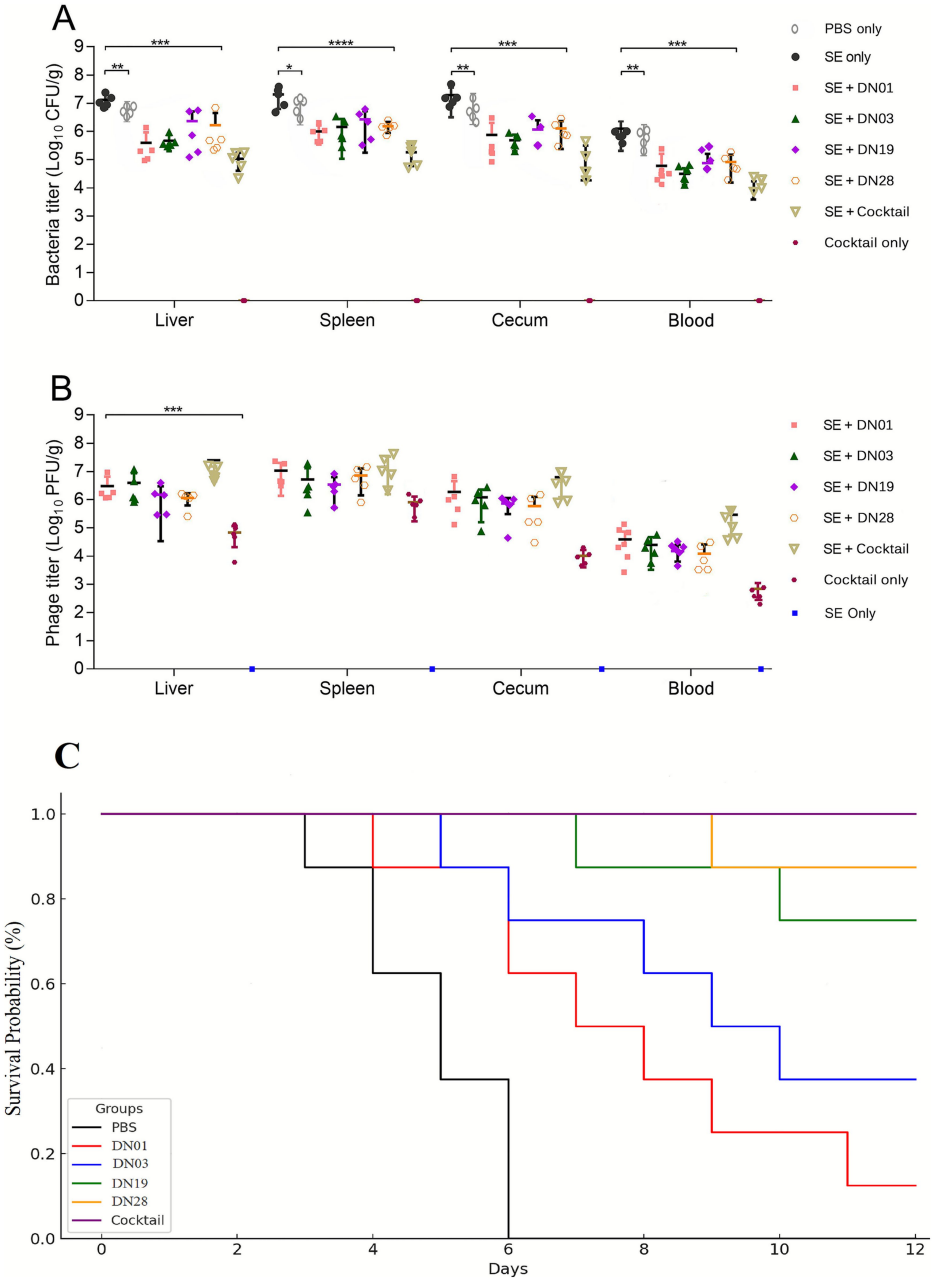
Therapeutic efficacy of individual *Salmonella* phages and phage cocktail in BALB/c mice infected with *S. enteritidis* SE006. **(A)** Bacterial loads (CFU/g) in blood, liver, spleen, and cecum 24 h post-treatment. **(B)** Corresponding phage titers (PFU/g) recovered from organ homogenates. **(C)** Kaplan–Meier survival curves over 12 days post-infection. Mice were treated with PBS (control), individual phages (DN01, DN03, DN19, DN28), or a phage cocktail (equal PFU of each phage, total ~1 × 10^10^ PFU/mL). Data represent mean ± SD (*n* = 8 per group). Survival differences were analyzed using the Log-rank (Mantel–Cox) test; *p* < 0.05. Phage-only mice (no infection) showed no adverse effects.

Phage therapy significantly reduced *Salmonella Enteritidis* loads in multiple mouse organs 24 h post-treatment (Figure 9A). The DN phage cocktail achieved superior bacterial clearance and exhibited higher *in vivo* replication compared to individual phages (Figure 9B). To confirm that these effects were attributable to active phages rather than toxicity, a phage-only (no bacterial infection) group was included, which showed no adverse effects or mortality, confirming the safety of phage administration *in vivo*.

The phage cocktail was prepared by mixing equal titers (1 × 10^10^ PFU/mL each) of the four phages (DN01, DN03, DN19, DN28). Prior to *in vivo* use, *in vitro* pairwise and combined assays confirmed the absence of antagonism and indicated potential synergistic interactions. The cocktail remained stable for at least 24 h at room temperature, retaining comparable lytic activity to the individual phages.

Mice (*n* = 8 per group) were intraperitoneally infected with ~1 × 10^8^ CFU of *S. enteritidis* SE006 and treated 3–4 h post-infection with individual phages or the cocktail. Bacterial loads in blood, liver, spleen, and cecum were significantly reduced (*p* < 0.05) in all phage-treated groups compared with PBS controls 24 h after treatment (Figure 9A). The phage cocktail group exhibited the most pronounced reduction, achieving nearly complete clearance in several organs. Correspondingly, phage titers recovered from organ homogenates were significantly higher in the cocktail group than in single-phage groups (Figure 9B), indicating robust *in vivo* replication and persistence.

Survival outcomes were monitored over 12 days using Kaplan–Meier analysis (Figure 9C). The PBS control group exhibited 0% survival by day 6. In contrast, survival rates in phage-treated groups improved as follows: DN01 (25%), DN03 (33%), DN19 (75%), DN28 (85%), and 100% in the cocktail-treated group. Statistical analysis using the Log-rank (Mantel–Cox) test confirmed significant differences among groups (*p* < 0.05). Mortality occurred earlier in the PBS and DN01 groups, whereas DN19-, DN28-, and cocktail-treated mice showed delayed or no mortality, highlighting the enhanced protective efficacy and durability of the phage cocktail.

### Histopathological assessment of cecal tissues following phage therapy in *Salmonella-*infected mice

**Figure 10:**
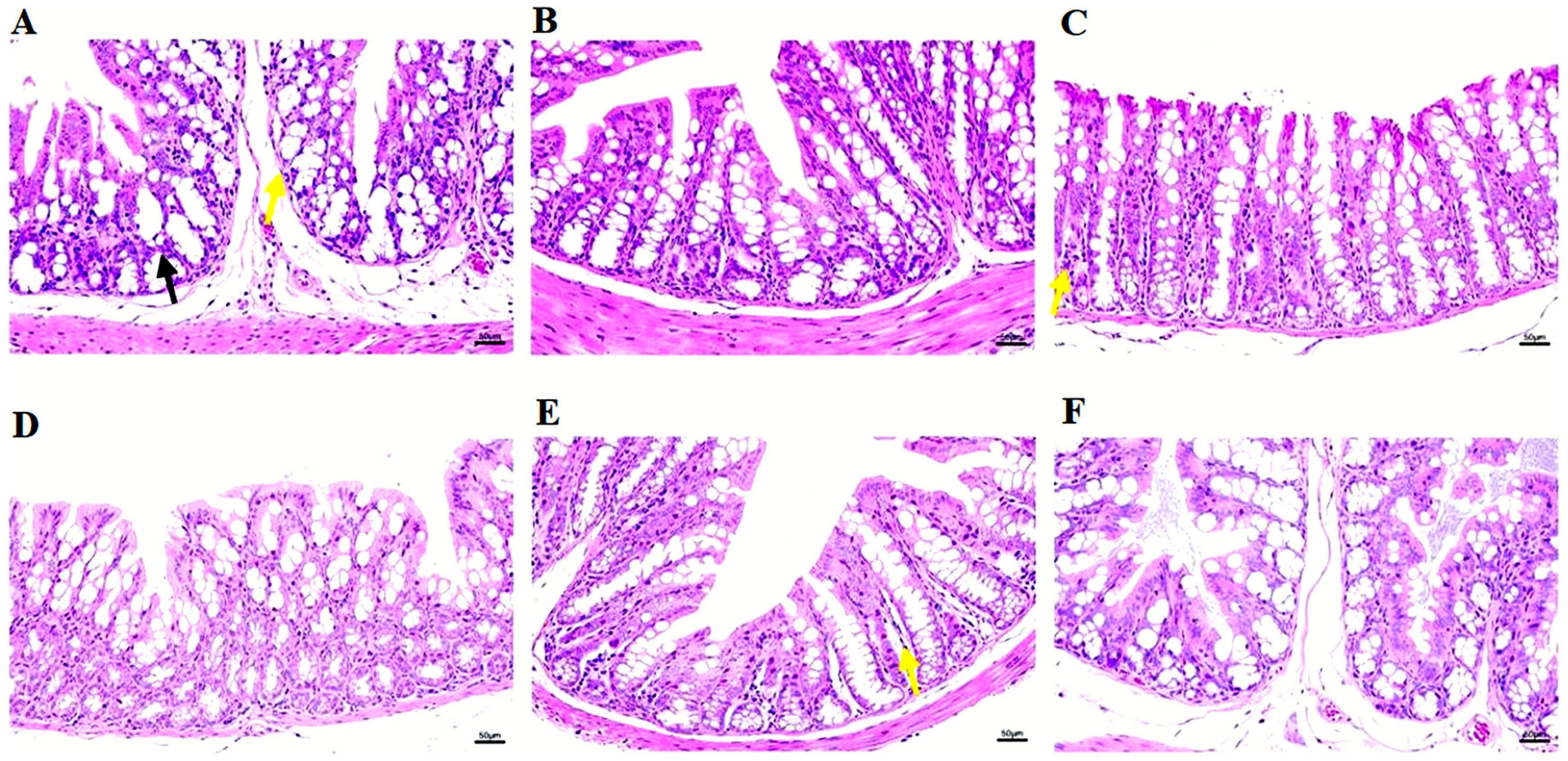
Pathological changes in the Cecum after 48 h. **(A)** PBS, **(B)** DN01, **(C)** DN03, **(D)** DN19, **(E)** DN28, **(F)** Cocktail. A-PBS was severely damaged; C-DN03 and E-DN28 were slightly damaged; the rest of the samples were normal. (B-DN01, D-DN19, F-Cocktail).

Cecal tissues from PBS-treated mice exhibited extensive damage, characterized by epithelial disruption and pronounced inflammation. In contrast, phage-treated groups, particularly those receiving DN01 and DN19, maintained near-normal tissue architecture with minimal pathological changes. Mild alterations were observed in DN01 and DN19 groups, whereas tissues from DN03, DN28, and the cocktail group appeared nearly intact (Figure 10).

## Discussion

This study presents a comprehensive characterization of four broad-host-range lytic *Salmonella* phages, DN01, DN03, DN19, and DN28, isolated from diverse environmental sources in China. These phages exhibited potent and specific lytic activity against multiple *Salmonella enterica* serovars, including multidrug-resistant (MDR) strains, underscoring their potential as alternatives to conventional antimicrobials. The observed broad lytic spectrum, particularly for DN03 and DN28, which lysed all tested strains, aligns with previous reports on polyvalent phages such as STP4-a and SHWT1 (19, 20). Such versatility is critical for overcoming the genetic diversity of clinical and environmental *Salmonella* isolates.

Morphological and genomic analyses placed these phages within the class Caudoviricetes, exhibiting structural features characteristic of the families Demerecviridae, Drexlerviridae, Autographiviridae and Straboviridae. Importantly, genome annotations revealed the absence of lysogeny-associated genes, virulence factors, and antibiotic resistance elements, meeting essential biosafety requirements for therapeutic use (15, 49). Their modular genomic organization, encoding replication, structural assembly, and lysis functions, supports a strictly lytic lifestyle and minimizes the risk of horizontal gene transfer.

The biological characterization demonstrated short latent periods, substantial burst sizes, and stability over a wide pH (3–11) and temperature range (4–60 °C), indicating high adaptability to diverse environments, including the gastrointestinal tract and food production settings (21). All four phages significantly inhibited biofilm formation and degraded established biofilms, an attribute of considerable clinical and industrial relevance, as biofilms contribute to chronic infections and reduced antibiotic efficacy (49, 50). Beyond controlled *in vitro* and *in vivo* experiments, phages have demonstrated substantial practical value in veterinary medicine and clinical applications. Phage therapy has been effectively utilized to reduce *Salmonella* colonization in poultry and livestock, minimize contamination in food products, and limit zoonotic transmission. In particular, phage cocktails provide broader efficacy by simultaneously targeting multiple *Salmonella* serovars, thereby mitigating the emergence of resistant strains and offering more durable protective effects. These translational applications underscore the practical potential of the characterized phages, reinforcing their significance not only for experimental research but also for real-world implementation in food safety management and animal health protection (Smith et al., 2021; Zhao et al., 2022; (13)).

*In vivo* therapeutic evaluation in a murine model of systemic *Salmonella* infection confirmed their efficacy, with all phage-treated groups showing markedly reduced bacterial burdens across multiple organs compared to untreated controls. Notably, the phage cocktail achieved complete survival over a 12-day observation period and superior bacterial clearance, likely due to synergistic host range expansion and enhanced *in vivo* replication. The delayed or absent mortality in cocktail-treated mice, compared with earlier deaths in monophage-treated groups, underscores the durability of protection. Similar findings in other enteric pathogens highlight the promise of cocktail formulations for reducing resistance emergence and extending therapeutic coverage (15, 20).

Collectively, these findings identify four genetically safe, environmentally stable, and phenotypically potent lytic phages with demonstrated *in vitro* and *in vivo* efficacy against multidrug-resistant *Salmonella enterica*. In addition to phage therapy, several alternative biocontrol strategies have been explored to counter antimicrobial resistance (AMR) in poultry production. Probiotic-based interventions particularly those utilizing *Bacillus* strains have demonstrated the ability to reduce *Salmonella* intestinal colonization and enhance host immunity (51). Similarly, β-glucans derived from indigenous yeast have shown protective effects against *Salmonella* infections in broilers by modulating immune responses and limiting bacterial persistence (52). Incorporating such bioactive and microbial-based approaches could complement phage therapy, offering a multi-pronged and sustainable strategy to mitigate multidrug-resistant *Salmonella* and improve food safety.

The integration of genomic, biological, and therapeutic evidence supports their development as promising agents for controlling *Salmonella* in veterinary and food safety contexts. Future work should focus on optimizing dosing regimens, evaluating host immune responses, and designing scalable delivery systems suitable for clinical and field applications, while ensuring biosafety and long-term environmental stability.

## Conclusion

This study reports the isolation and characterization of four novel lytic bacteriophages (DN01, DN03, DN19, DN28) with broad host ranges and potent lytic activity against *Salmonella enterica* SE006. Although this strain was not tested for antibiotic resistance in the current study, it represents a clinically relevant *Salmonella* population that may include antibiotic-resistant isolates. The phages exhibited high environmental stability, anti-biofilm efficacy, and were devoid of undesirable genes. *In vivo* experiments confirmed their therapeutic efficacy and safety in a murine infection model. Collectively, these findings highlight the potential of these phages as promising biocontrol agents for managing *S. enterica* infections, including those caused by multidrug-resistant strains.

## Data Availability

The original contributions presented in the study are publicly available. This data can be found here: https://doi.org/10.6084/m9.figshare.30621803.

## Footnotes

1
https://rast.nmpdr.org/

2
https://blast.ncbi.nlm.nih.gov/Blast.cgi

3
https://cge.cbs.dtu.dk/services/ResFinder/

4
https://cge.cbs.dtu.dk/services/VirulenceFinder/
